# Lesion probability mapping in MS patients using a regression network on MR fingerprinting

**DOI:** 10.1186/s12880-021-00636-x

**Published:** 2021-07-08

**Authors:** Ingo Hermann, Alena K. Golla, Eloy Martínez-Heras, Ralf Schmidt, Elisabeth Solana, Sara Llufriu, Achim Gass, Lothar R. Schad, Frank G. Zöllner

**Affiliations:** 1 Computer Assisted Clinical Medicine, Medical Faculty Mannheim, Heidelberg University, Mannheim, Germany; 2 Department of Imaging Physics, Delft University of Technology, Delft, Netherlands; 3 Mannheim Institute for Intelligent Systems in Medicine, Medical Faculty Mannheim, Heidelberg University, Mannheim, Germany; 4 Center of Neuroimmunology, Laboratory of Advanced Imaging in Neuroimmunological Diseases, Hospital Clinic Barcelona, Institut d’Investigacions Biomédiques August Pi i Sunyer (IDIBAPS), Universitat de Barcelona, Barcelona, Spain; 5 Department of Neurology, Medical Faculty Mannheim, Heidelberg University, Mannheim, Germany

**Keywords:** Deep learning reconstruction, Magnetic resonance fingerprinting, Lesion prediction, $T_1$ Mapping, ${T_2}^*$ Mapping

## Abstract

**Background:**

To develop a regression neural network for the reconstruction of lesion probability maps on Magnetic Resonance Fingerprinting using echo-planar imaging (MRF-EPI) in addition to $T_1$, ${T_2}^*$, NAWM, and GM- probability maps.

**Methods:**

We performed MRF-EPI measurements in 42 patients with multiple sclerosis and 6 healthy volunteers along two sites. A U-net was trained to reconstruct the denoised and distortion corrected $T_1$ and ${T_2}^*$ maps, and to additionally generate NAWM-, GM-, and WM lesion probability maps.

**Results:**

WM lesions were predicted with a dice coefficient of $0.61\pm 0.09$ and a lesion detection rate of $0.85\pm 0.25$ for a threshold of 33%. The network jointly enabled accurate $T_1$ and ${T_2}^*$ times with relative deviations of 5.2% and 5.1% and average dice coefficients of $0.92\pm 0.04$ and $0.91\pm 0.03$ for NAWM and GM after binarizing with a threshold of 80%.

**Conclusion:**

DL is a promising tool for the prediction of lesion probability maps in a fraction of time. These might be of clinical interest for the WM lesion analysis in MS patients.

**Supplementary Information:**

The online version contains supplementary material available at 10.1186/s12880-021-00636-x.

## Background

Assessment and segmentation of white matter (WM) lesions is an important step for the analysis and tracking of diseases such as multiple sclerosis (MS). WM lesions can be graded based on MRI images which showed a good correlation with symptom development in MS and clinical subtypes of MS. [1, 2] Lesion probability mapping is a method to differentiate between WM lesion groups as this corresponds to different ischemic components and neurodegeneration during disease progression. [3–6] Additionally, WM lesions exhibit an increased $T_1$, $T_2$, and ${T_2}^*$ relaxation time, and therefore, multiple quantitative approaches showed advantages in the detection, grading, and classification. [7–9] In particular, Magnetic Resonance Fingerprinting (MRF) has demonstrated a variety of applications for simultaneously quantifying multiple relaxation times at clinically acceptable scan times. In conventional MRF, thousands of highly undersampled images are acquired to produce a unique fingerprint, and these fingerprints are compared voxel-wise with a pre-calculated dictionary. [10, 11] Rieger et al. proposed an MRF method to quantify $T_1$ and ${T_2}^*$ with an echo-planar imaging (EPI) readout, [12] which showed promising results in renal and neural applications. [13–16] The fact that only conventional undersampling factors lead to only slightly corrupted magnitude data reduces the time for reconstruction and increases its robustness. However, a major drawback of MRF is the tradeoff between reconstruction time and accuracy.

Deep learning (DL) has emerged into the field of MRI and achieved excellent results in data processing considering accuracy, precision, and speed. Hence, DL is increasingly outperforming conventional algorithms. Previous studies and reports suggest that convolutional neural networks (CNN) can solve high dimensional problems with excellent accuracy and in a short time for denoising, distortion correction, segmentation, classification, and reconstruction. [17–23] A promising architecture is the U-net, which has great diversity for applications such as segmentation and regression tasks. [24–26] Especially in MRF, the reconstruction of the enormous amount of acquired data can be improved and accelerated by using different network architectures such as CNN’s and fully convolutional networks. [27–32] In previous work, a CNN was used for the denoising, distortion correction, reconstruction, and generation of NAWM and gray matter (GM) probability maps yielding results comparable to conventional methods in a fraction of time. [16] The proposed architecture combined several post-processing tasks, making the application fast and easy. However, the WM lesions have to be segmented for further analysis, which is always time-consuming and suffers from high intra- and inter-observer variabilities. [33] To overcome these limitations of manual segmentation, different DL architectures and networks have been used, yielding dice coefficients ranging from 0.48 to 0.95 for WM lesion segmentation. [33–36] Therefore, in a recent publication, it was shown that the use of regression networks for generating distance maps of the lesions might improve the WM lesion segmentation process [37]. This could provide more information about lesion geometry, structure, and changes similar to lesion probability mapping. [2, 3, 38]

In this work, we use the U-net as previously reported [16] to predict WM lesion probability maps by training the CNN with the manual annotated binary lesion masks while combining several processing steps.

## Methods

### Data

As previously reported [16], an MRF sequence based on echo-planar imaging was acquired across 6 healthy subjects and 18 patient with WM lesions at a 3T scanner (Magnetom Skyra, Siemens Healthineers; site 1) and 24 patient with WM lesions at a 3T scanner (Magnetom Prisma, Siemens Healthineers; site 2). The sequence parameters for both scanners were FOV = 240 $\times$ 240 mm, in-plane resolution = $1\times 1\mathrm{mm}^2$, slice thickness = 2 mm, GRAPPA factor = 3, partial Fourier = 5/8, varying flip angle $\alpha$ (34–86$^\circ$), TE (16–76.5 ms), TR (3530–6370 ms). At site 2, simultaneous multi-slice imaging was additionally used with an acceleration factor of 3.

### CNN

**Fig. 1:**
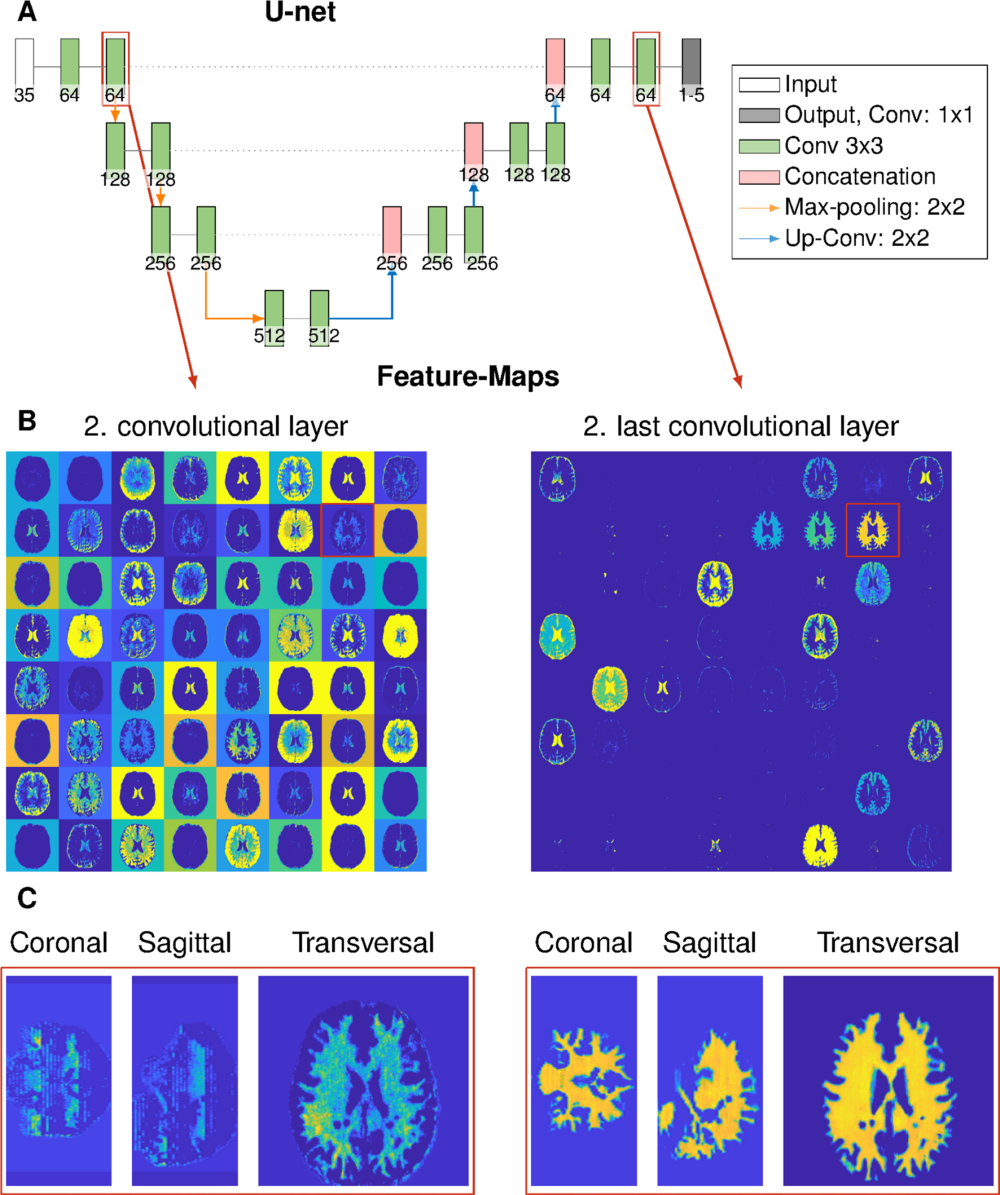
**a** Representation of the U-net with an encoder depth of three. **b** Feature maps of the second convolutional layer and the second last convolutional layer are depicted. One feature per layer is marked in red and shown below in **c**. Coronal, sagittal, and transversal slices of the corresponding features maps are shown. The second convolutional layer shows a WM-like feature, which, however, is not homogeneous in all three dimensions. The last convolution layer depicts a homogeneous WM-like feature in all three dimensions. No colorbars were shown because all features are in arbitrary units

A U-net (Fig. 1a) was used for the denoising, distortion correction, and reconstruction of $T_1$, ${T_2}^*$ maps, and the NAWM-, GM-, and additionally lesion probability maps. The $T_1$ and ${T_2}^*$ maps for training the network were reconstructed using pattern matching with a precision of 5% for the variety of $T_1$ (300–3500 ms), ${T_2}^*$ (10–2500 ms) times, and the flip angle scale factor (0.6–1.4, in steps of 0.1) to correct for $B_1^+$ inhomogeneities. Beforehand, denoising was applied using Marchenko–Pastur Principal Component Analysis (MPPCA) [39]. The dictionary entries are in steps of 5%. Rigid registration was performed using B-spline interpolation from the undistorted $T_1$ map to the in this protocol additionally acquired $T_2$-weighted image using the Advanced Normalization Tools (ANT). [40] The NAWM and GM maps were generated based on the distortion corrected $T_1$ maps using SPM (Statistical Parametric Mapping) [41] with a probability between 0 and 100%. Additionally, WM lesions were segmented manually by an expert radiologist on the FLAIR data, and to assess the intra-observer variability, lesions from ten patients were segmented two times (at least one week time gap), and the mean dice coefficient was calculated. The manually annotated binary lesion masks were used as a fifth training output of the CNN. The training input was always the 35 magnitude MRF-EPI data. We used two patients from site 1 and three patients from site 2 as test data and the same amount as validation data. We trained our network patch-wise using 64 random patches per slice with a patch size of $64 \times 64$ voxels, a mini batch-size of 64, 100 training epochs, and a learning rate of $10^{-4}$. Slices containing white matter lesions with a minimum volume of 100 ml were augmented by a factor of five to overcome the small overall volume of the lesions compared to the whole brain. We trained our networks using four different loss functions (MAE, MSE, LCL) with all five output maps ($T_1$, ${T_2}^*$, NAWM-, GM-probability maps, and WM lesion masks) and additionally the other networks using DICE loss with only the lesion masks as output for comparison with the conventional lesions segmentation. Additionally, the U-net was trained for the network number 8 with the denoised and distortion corrected $T_1$ and ${T_2}^*$ maps and the lesion mask as output for comparison to conventional DL processing (MSE-2-1). The following loss functions were used for one and five outputs: mean squared error (MSE), mean absolute error (MAE), logarithmic cosinus hyperbolic loss (LCL), and dice loss (DICE) as listed in Table 1. The naming MSE-1 and MSE-5 correspond to the loss function with the number of outputs.

**Table 1:** All network architectures are listed which are used in this manuscript.

Network	Loss	Inputs	Outputs	Naming
1	MSE	35 (MRF baseline)	5 ($T_1$, ${T_2}^*$, WM-, GM-, lesion prob. maps)	MSE-5
2	MAE	35 (MRF baseline)	5 ($T_1$, ${T_2}^*$, WM-, GM-, lesion prob. maps)	MAE-5
3	LCL	35 (MRF baseline)	5 ($T_1$, ${T_2}^*$, WM-, GM-, lesion prob. maps)	LCL-5
4	MSE	35 (MRF baseline)	1 (lesion prob. map)	MSE-1
5	MAE	35 (MRF baseline)	1 (lesion prob. map)	MAE-1
6	LCL	35 (MRF baseline)	1 (lesion prob. map)	LCL-1
7	DICE	35 (MRF baseline)	1 (lesion prob. map)	DICE-1
8	MSE	2 ($T_1$, ${T_2}^*$ map)	1 (lesion prob. map)	MSE-2-1

Networks 3, 5, 6, 7 were not converging into lesion probability maps

The loss functions mean absolute error (MAE), mean squared error (MSE), locarithmic hyperbolic cosinus loss (LCL), and dice loss (DICE) are used. The number of outputs is either 5 ($T_1$, ${T_2}^*$ maps and NAWM-, GM-, and lesion probability maps) or 1 (only lesion probability map)

For all loss functions, the network was trained with both the five output maps and also only the lesion as output to validate the loss in accuracy when using multiple outputs. In previous work, it was observed that for multiple outputs the accuracy decreases of the network. [16] The accuracy compared with the conventional methods was validated with the MSE-5 because this was the best architecture for the reconstruction of $T_1$, ${T_2}^*$, NAWM-, and GM-probability maps and lesion probability maps.

### Statistics

The Dice coefficient and the lesion detection rate were used as the similarity metric for the lesion segmentation. Therefore, the threshold for binarizing the reconstructed lesion probability maps was analyzed. NAWM and GM masks were binarized with a commonly used threshold of 80% [42] and mean dice coefficients along all subjects and slices were calculated. For the two other outputs ($T_1$, ${T_2}^*$) the mean relative difference was calculated.

## Results

The reconstruction with DL showed good agreement with conventional pattern matching reconstruction and a mean relative deviation of 5.2% for $T_1$ and 5.1% for ${T_2}^*$ in the whole brain using MSE-5. The Dice coefficients for NAWM and GM after binarization with a threshold of 80% were $0.92\pm 0.04$ for NAWM and $0.91\pm 0.03$ for GM using MSE-5. The reconstruction of all five outputs took around one minute for the whole brain per subject, which is several orders of magnitude faster compared to the conventional processing (denoising, MRF reconstruction, distortion correction, masking, and lesion segmentation) of about three hours.

**Fig. 2:**
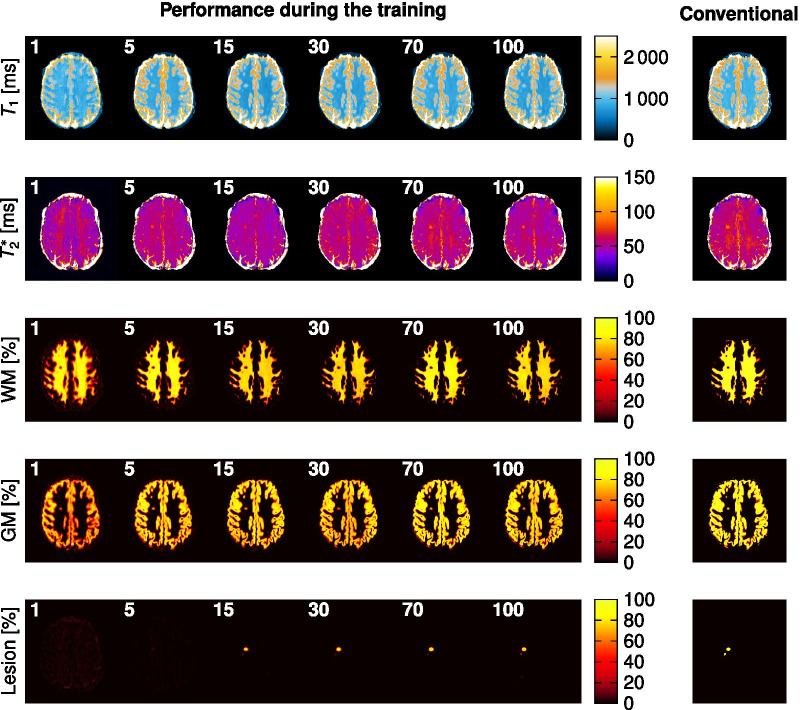
Visualization of the reconstruction during the training. The reconstructed $T_1$, ${T_2}^*$, NAWM-, GM, and Lesion-probability maps are depicted for 1, 5, 15, 30, 70, and 100 training epochs (white number) and the dictionary matching reference maps are shown on the right side for MSE-5

Figure 2 shows the $T_1$, ${T_2}^*$, NAWM-, GM-, and lesion probability maps generated by the CNN (MSE-5) for different training epochs (1, 5, 15, 30, 70, 100) compared to the conventionally reconstructed maps and the segmented masks. Visual good image quality was obtained for $T_1$, ${T_2}^*$, NAWM-, and GM probability maps after already 5 epochs. After around 15 epochs, the network starts to predict the lesion probability maps and slowly converges towards 100 epochs.

**Fig. 3:**
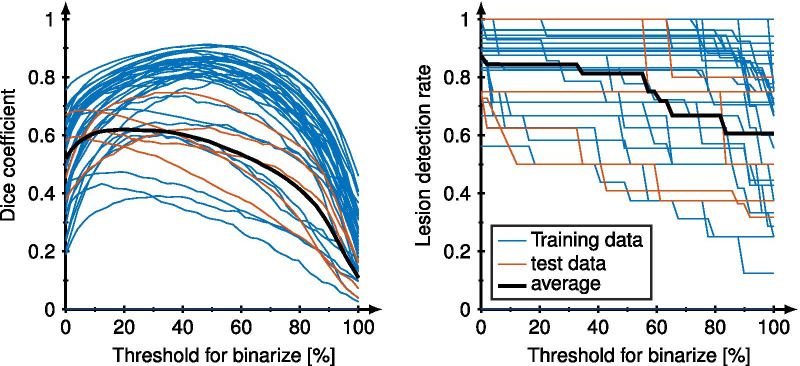
The dice coefficient (left) and the lesion detection rate (right) for all training data (blue) and test data (orange) are shown over the threshold to binarize the lesion probability maps. The black lines depict the average across the test data. A maximum dice coefficient is observed at a threshold of around 50%. The lesion detection rate decreases for an increasing threshold because the background of the lesion probability map is non-zero

The dice coefficient was strongly dependent on the threshold for binarizing the probability maps which is shown in Fig. 3. A maximum dice coefficient of 0.75 is observed for a threshold of 41% for the training data (depicted in blue) and a maximum dice coefficient of 0.62 for a threshold of 23% for the test data (depicted in orange) respectively. For further analysis, a threshold of 33% was used to binarize the lesion probability maps into masks as the best compromise for both, training and test data. At lower thresholds, the lesion detection rate increases. The dice coefficient and the lesion detection rate were $0.61\pm 0.09$ and $0.85\pm 0.25$ for the test data using the threshold of 33%. The average dice coefficient with its intra-observer variability across different annotations was $0.68\pm 0.15$. After training the network with this second set of annotations the dice coefficient and the lesion detection rate were $0.60\pm 0.17$ and $0.84\pm 0.19$. The prediction of the WM lesions does not depend on the lesion volume as illustrated in Additional file 1: Figure S1.

**Fig. 4:**
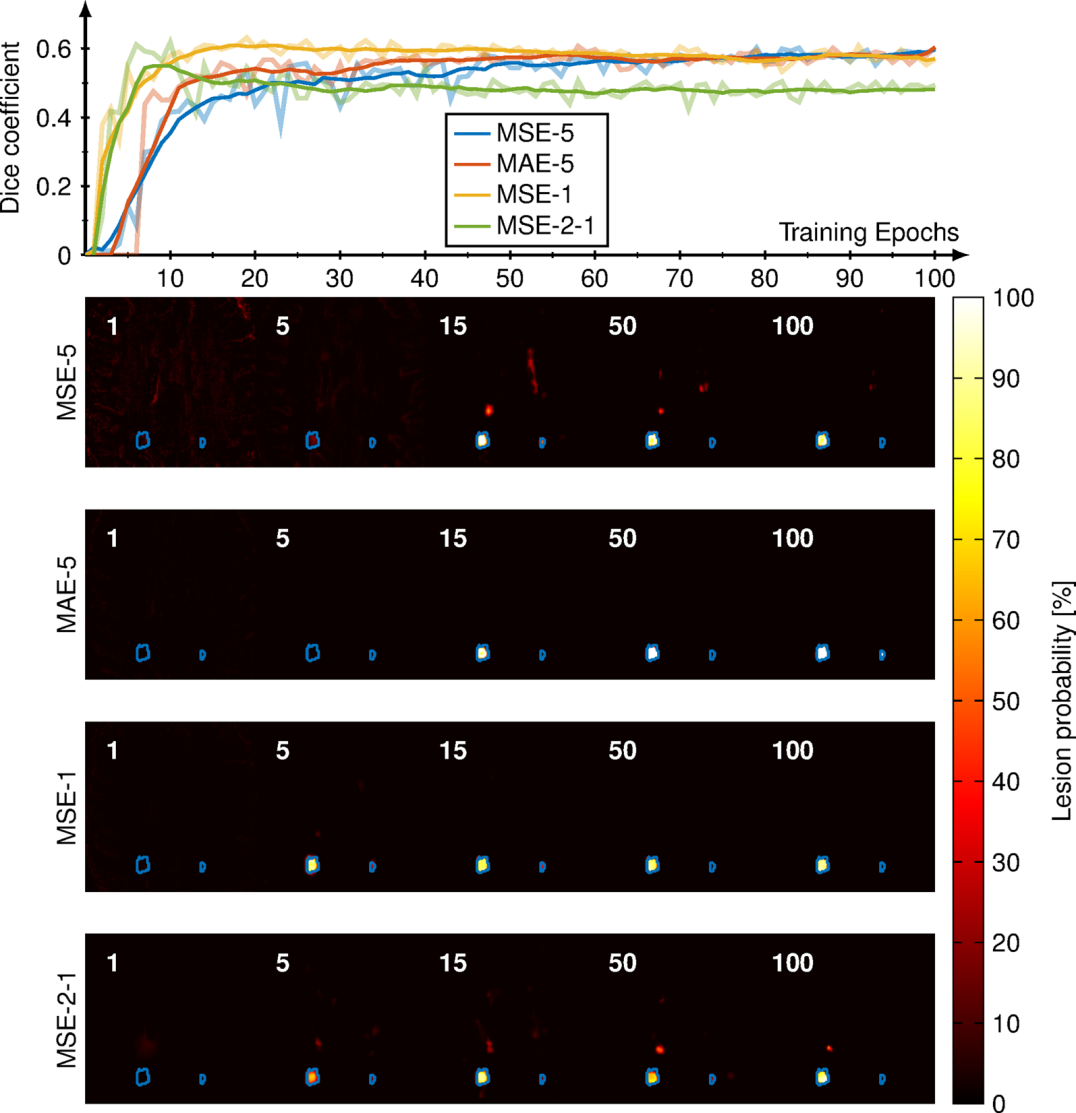
The dice coefficient for three different networks is depicted (five outputs with MSE [MSE-5], five outputs with MAE [MAE-5], only lesions with MSE [MSE-1]) and the reference network with the $T_1$ and ${T_2}^*$ maps as input and lesions as output [MSE-2-1]. The dice coefficient is plotted for all three networks over the training epochs and the smoothed data is shown in the foreground colors. The corresponding lesion probability maps are shown for 1, 5, 15, 50, and 100 epochs below

In Fig. 4, the lesion probability is plotted versus the number of training epochs for different networks. It can be seen that training only with 1 output instead of 5 results in faster convergence of the dice coefficient, however, the dice coefficient for the three methods (MSE-5, MAE-5, MSE-1) converged to 0.61 after about 60 epochs. The reference network (MSE-2-1) reached after around 7 epochs the maximum dice coefficient of 0.61 but then decreases towards 0.5. The mean lesion detection rate over the entire test data was 0.85 for MSE-5, 0.79 for MAE-5, 0.78 for MSE-1, and 0.73 for MSE-2-1. The training with MAE-5 takes longer to start predicting lesions. The networks MAE-1, LCL-5, LCL-1, and DICE-1 converge to a local minimum while training, resulting in all lesion probabilities equal to zero and were therefore excluded from the analysis.

**Fig. 5:**
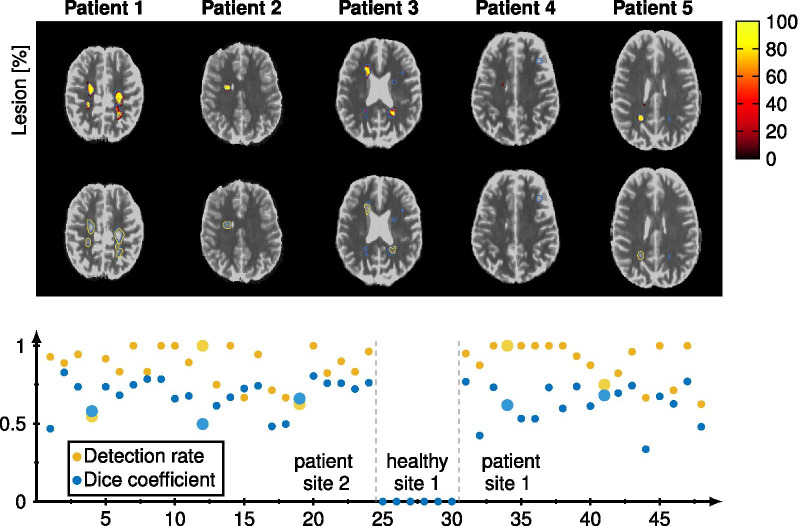
The reconstructed lesions probability maps are overlayed on the magnitude data in color encoding for all five different patients from the test set. Manual annotation is depicted in blue. Below the probability map is binarized and depicted in yellow in addition. The dice coefficient and white matter lesion detection rate is depicted for every patient and healthy subject for both sites. The average lesions detection rate is 0.88 and the average dice coefficient is 0.67 for all patients. The test data is shown in larger marks and brighter color and yields an average lesion detection rate of 0.85 and an average dice coefficient of 0.61 using the MSE-5

For every test patient, one representative slice is shown in Fig. 5 with the lesion probability color-encoded, and the manual annotation highlighted in blue. For patients number 1, 2, and 5 the depicted lesions of the slice correlate very well with the annotation. For patient number 3, the CNN predicted three lesions with a small probability, which were then excluded from the mask after thresholding. Only in test patient number 4, the network did not predict the annotated lesion near the GM. The dice coefficient and lesion detection rate are shown for all subjects in Fig. 5. The test data are shown in larger marks with lighter blue and yellow colors. The CNN predicted no lesions in healthy subjects.

**Fig. 6:**
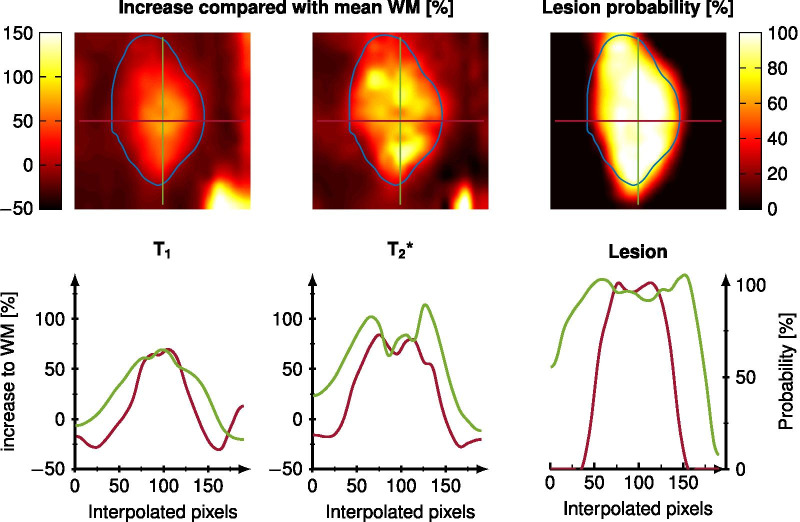
One lesion is depicted in a zoomed-in version with a bilinear interpolation of factor 10. The increase in $T_1$ and ${T_2}^*$ compared with the mean NAWM is color encoded in percentage and the lesion probability generated by the CNN is shown on the right side. The manual annotation is drawn as a blue line. Below the voxel-wise values are depicted for one horizontal (red) and one vertical (green) cut through the lesion

Figure 6 shows the percentage increase of a WM lesion compared to the mean NAWM times for $T_1$, ${T_2}^*$, and the lesion probability generated by the CNN. The manually annotated lesion is marked in blue. A good visual correlation between the lesion probability and the increase in $T_1$ and ${T_2}^*$ is observed, as depicted below for the two cross-sections (green and red). It was also observed that the lesion probability is increased and steeper for lesions that have increased relaxation times.

## Discussion

In this study, we have shown that the CNN is capable of predicting lesion probability maps, which correlate with an increase in $T_1$ and ${T_2}^*$ times in NAWM. After binarizing the probability maps, the dice coefficient was $0.61\pm 0.09$ for the test data, which is comparable to the intra-observer variability of the manual drawer ($0.68\pm 0.23$) and is comparable to literature (0.47–0.95). [33, 36] The CNN might be more robust compared with manual annotations because the network has no variations for multiple annotations. We have shown that the network only predicts lesion probability maps for the loss functions MAE and MSE. This could be since outliers, such as the small spherical lesions, are weighted more heavily with MSE and MAE compared with LCL or the dice loss. This was also observed for MAE-1, despite MAE-5 was able to predict lesions. More augmentation or an advanced optimization of the network considering the layer structure could further improve the performance of the network. In each case, training with one or all five output masks converged to the same dice coefficient regardless of the network, demonstrating the ability to reconstruct all maps within a single architecture (Fig. 4). Compared to the conventional method of performing lesion segmentation on the $T_1$ and ${T_2}^*$ maps, the network MSE-2-1 resulted in an average dice of 0.5 and yielding worse performance compared with the other proposed networks. With this, we state that the approach of combining all post-processing steps has no loss in accuracy when lesion probability maps are generated facilitating an advanced DL approach to save time and avoid further processing.

Additionally, the network was able to perform the tasks of reconstruction, denoising, distortion correction, and segmentation within a single architecture with promising accuracy. $T_1$ and ${T_2}^*$ maps as well as the NAWM- and GM-probability maps showed good agreement as also previously reported [16] with a mean relative error of 5.2% for $T_1$ and ${T_2}^*$ and mean dice coefficients of higher than 0.9 for NAWM and GM. The use of a single network is advantageous due to the faster and simpler reconstruction compared with several networks for the different processing steps although the accuracy seemed to be slightly compromised. [16] It was observed, that the network first learns to reconstruct the $T_1$, ${T_2}^*$, NAWM, and GM probability maps, as evidenced by the good visual image quality after only 5 epochs (Fig. 2). This could be explained by the several orders increase in the number of non-zero voxels in these maps compared to the low number of lesion voxels per slice.

The lesion probability maps visually correlate well with the increase in $T_1$ and ${T_2}^*$ compared to the mean NAWM times. This could indicate that larger or more intense lesions are also predicted as such by the CNN. Therefore, these lesion probability maps could be used to automatically rate and differentiate different lesions based on the MRF input data. This is similar to the results of other lesion probability mapping methods. However, these methods rely either on manual grading, voxel-wise, or local spatial dependent models, which are time-consuming and susceptible to patient-specific covariances. [1–3, 5] A large cohort is beneficial to prove this assumption and to correlate this behavior with follow-up measurements and disease specificities.

Besides, our approach could include the underlying information of the evolution of the MRF scan. It has been shown that principal component analysis (PCA), which also uses the input magnitude MRF data, allows separation of the brain into multiple components such as myelin and WM lesions [43, 44]. This is shown in Fig. 1b where all feature maps of the second and second last convolutional layer are depicted. At the beginning of the network architecture, features from the MRF magnitude images are extracted. They are not homogeneous along all three dimensions as also the magnitude MRF data are not. However, in subsequent layers, the feature maps are homogeneous in 3 dimensions meaning that anatomical features independently on the slice position and acquisition scheme are extracted. The CNN might be able to learn and distinguish the underlying components, improving lesion segmentation and prediction. This is an information gain compared to manual annotators and compared to lesion segmentation methods based solely on the quantitative parametric maps. [36, 37] In further work, this has to be compared with conventional methods for assessing these components such as the myelin or extracellular water. [43, 45, 46]

This study has some limitations. Because the lesions were manually segmented, there is a large amount of variation in the annotation, which was also evident in the relatively high intra-observer variability (0.68). This could be improved by performing more annotations from multiple annotators to reduce this variability, but this is very time-consuming. The reduced variability in the lesion masks could also lead to better and faster training performance of the network, yielding higher dice coefficients. WM lesions are often difficult to differentiate from NAWM in the $T_1$ and ${T_2}^*$ maps without knowledge of surrounding layer information because NAWM lesions appear similar to lobes of the GM inside the NAWM. The reconstruction could be improved by using a 3D CNN with 3D patches. However, we have tried to train a 3D architecture, but the 3D CNN was not able to predict any lesions and the accuracy for the other outputs was compromised. This could be because 3D architectures require more data and longer training compared with 2D CNNs. Therefore, more data needs to be acquired for comparable 3D results, which will be the content of further work. This could also be the reason why some loss functions could not generate lesion probability maps.

## Conclusion

In this work we showed, that training a neural network with lesion masks can be used to generate lesion probability maps, which might improve diagnostics. Additionally, the single CNN is a promising tool for the reconstruction, denoising, distortion correction of $T_1$ and ${T_2}^*$ maps, and additionally to generate NAWM, GM probability maps. The reconstruction for a whole brain took less than one minute, which is more than a 100 fold acceleration compared with conventional processing which makes it clinically of great interest.

## Supplementary Information

**Additional file 1**. **Figure S1**: A) Dice coefficient per lesion is depicted over the lesion volume inml in a logarithmic scale towards x. For every single lesion the dice coefficient was calculated between the lesion masks annotated manually and using the CNN. In blue the training data is shown and in orange the test data. B) Predicted lesion volume is plotted over the true lesion volume in ml in double logarithmic scale. It shows a linear dependency close to the bisector depicted in gray. **Table S1**: All network architectures are listed which are used in this manuscript.The loss functions mean absolute error (MAE), mean squared error (MSE), locarithmic hyperbolic cosinus loss (LCL), and dice loss (DICE) are used. The number of outputs is either 5 (T_1_, T_2_^*^ maps and NAWM-, GM-, and lesion probability maps) or 1 (only lesion probability map).

## Data Availability

The Matlab scripts for generating and training the U-net are available here: https://github.com/PhysIngo/Lesion-Probability-Mapping.git. The datasets used and/or analyzed during the current study are available from the corresponding author on reasonable request.

## Abbreviations

WM: White matter
NAWM: Normal appearing white matter
MS: Multiple sclerosis
MRI: Magnetic resonance imaging
MRF: Magnetic resonance fingerprinting
EPI: Echo planar imaging
DL: Deep learning
CNN: Convolutional neural network
GM: Gray matter
MPPCA: Marchenko–Pastur principle component analysis
ANT: Advanced normalization tools
SPM: Statistical parametric mapping
FLAIR: Fluid attenuated inversion recovery
MAE: Mean absolute error
MSE: Mean squared error
LCL: Logarithmic cosinus loss

## Declarations

### Ethik-Kommission site 1 (2019-711N)

Medizinische Fakultít Mannheim der Ruprecht-Karls-Universitít Heidelberg z. Hd. Songhui Cao/Manja Goerner/Kathrin Heberlein Zentrum Medizinischer Forschung Haus 42, 3. OG Theodor-Kutzer-Ufer 1-3 68167 Mannheim, Germany

### Ethik-Kommission site 2 (BCB2012/7965)

Secretario del Comité Ético de Investigación Clínica del Hospital Clínic de Barcelona. Joaquim Forés I Vineta, Andrea Scalise, Ana Lucia Arellano Andrino, Hospital Clinic, calle Villarroel 170 08036 Barcelona, Spain.
