# Supplementary material for: Lesion probability mapping in MS patients using a regression network on MR fingerprinting
Source: BMC Med Imaging. 2021 Jul 8;21:107. doi: 10.1186/s12880-021-00636-x (PMC8265034; doi:10.1186/s12880-021-00636-x)
Supplement: Supplementary file 1 — Additional file 1. Figure S1: A) Dice coefficient per lesion is depicted over the lesion volume inml in a logarithmic scale towards x. For every single lesion the dice coefficient was calculated between the lesion masks annotated manually and using the CNN. In blue the training data is shown and in orange the test data. B) Predicted lesion volume is plotted over the true lesion volume in ml in double logarithmic scale. It shows a linear dependency close to the bisector depicted in gray. Table S1: All network architectures are listed which are used in this manuscript.The loss functions mean absolute error (MAE), mean squared error (MSE), locarithmic hyperbolic cosinus loss (LCL), and dice loss (DICE) are used. The number of outputs is either 5 (T1, T2* maps and NAWM-, GM-, and lesion probability maps) or 1 (only lesion probability map). [file 12880_2021_636_MOESM1_ESM.pdf]

SUPPLEMENTARY MATERIAL

The supplementary material is for both review and online publication. It is listed in numerically below with corresponding captions.

SUPPLEMENTARY FIGURES

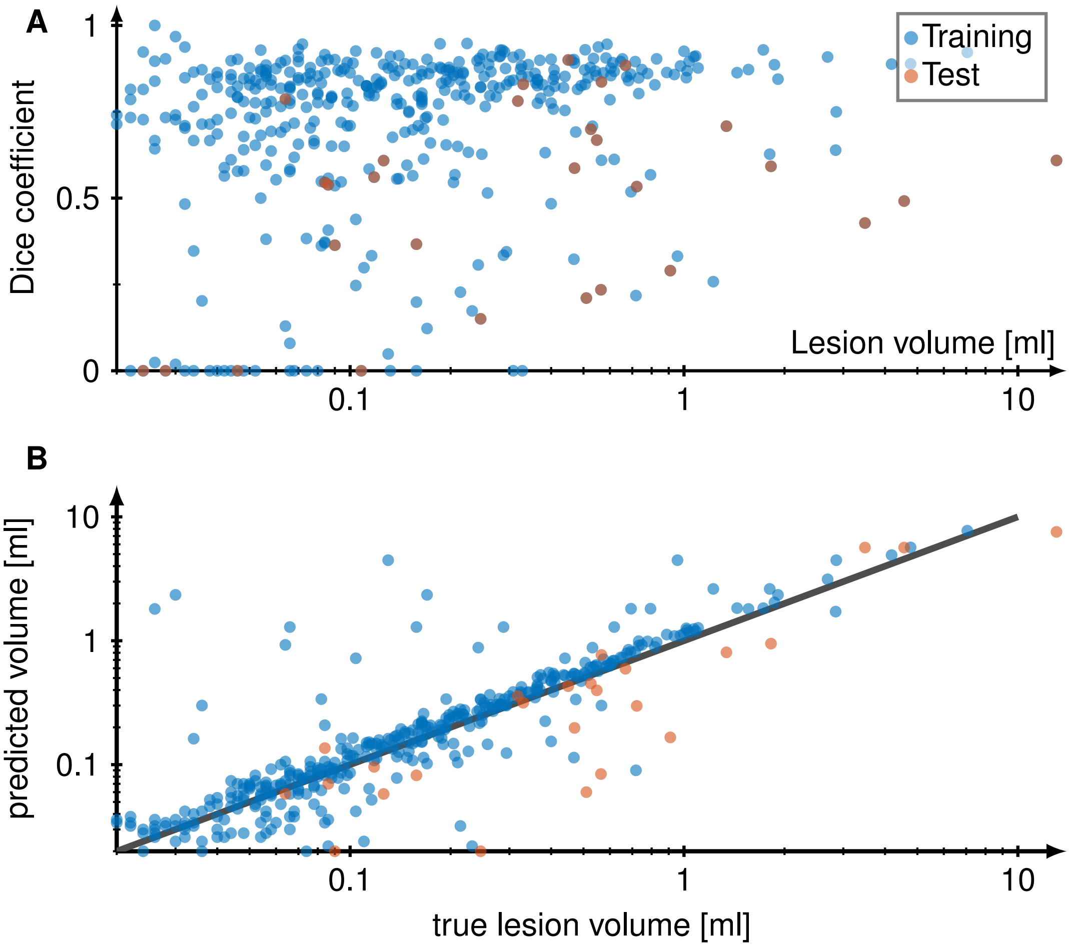

Supporting Information Figure S 1: A) Dice coefficient per lesion is depicted over the lesion volume in ml in a logarithmic scale towards x. For every single lesion the dice coefficient was calculated between the lesion masks annotated manually and using the CNN. In blue the training data is shown and in orange the test data. **B)** Predicted lesion volume is plotted over the true lesion volume in ml in double logarithmic scale. It shows a linear dependency close to the bisector depicted in gray.

SUPPLEMENTARY TABLES

Supporting Information Table S 1: All network architectures are listed which are used in this manuscript. The loss functions mean absolute error (MAE), mean squared error (MSE), locarithmic hyperbolic cosinus loss (LCL), and dice loss (DICE) are used. The number of outputs is either 5 (T_1, T_2^* maps and NAWM-, GM-, and lesion probability maps) or 1 (only lesion probability map).

network	loss	outputs	naming
1	MSE	5	MSE-5
2	MAE	5	MAE-5
3	LCL	5	LCL-5
4	MSE	1	MSE-1
5	MAE	1	MAE-1
6	LCL	1	LCL-1
7	DICE	1	DICE-1
